# Anthropometric features as predictors of atherogenic dyslipidemia and cardiovascular risk in a large population of school-aged children

**DOI:** 10.1371/journal.pone.0197922

**Published:** 2018-06-01

**Authors:** José M. Furtado, Sílvia M. Almeida, Paulo Mascarenhas, Maria E. Ferraz, José C. Ferreira, Manuel Vilanova, Mariana P. Monteiro, Fernando P. Ferraz

**Affiliations:** 1 Centro de Genética Médica e Nutrição Pediátrica Egas Moniz, Campus Universitário, Monte da Caparica, Portugal; 2 Instituto Universitário Egas Moniz, Campus Universitário, Monte da Caparica, Portugal; 3 Department of Obstetrics and Gynecology of University of Medicine of Warsaw, Warsaw, Poland; 4 Instituto de Investigação e Inovação em Saúde, and IBMC–Instituto de Biologia Molecular e Celular, Universidade do Porto, Porto, Portugal; 5 Instituto de Ciências Biomédicas de Abel Salazar, Universidade do Porto, Porto, Portugal; 6 Clinical and Experimental Endocrinology Group, Unit for Multidisciplinary Research in Biomedicine UMIB, ICBAS, University of Porto, Porto, Portugal; Universidad Miguel Hernandez de Elche, SPAIN

## Abstract

**Background:**

Autopsy studies reveal that atherosclerosis lesions can be found as early as two years of age. To slow the development of this early pathology, obesity and dyslipidemia prevention should start from childhood making it urgent to explore new ways to evaluate dyslipidemia risk in children that can be applied widely, such as the non-invasive anthropometric evaluation.

**Objective:**

Assess the metabolic profile of a pediatric population at a specific age to describe the association between anthropometric and biochemical cardiovascular disease risk factors; and evaluate selected anthropometric variables as potential predictors for dyslipidemic cardiovascular risk.

**Design and methods:**

Anthropometric features, bioimpedance parameters and fasting clinical profile were assessed in Lisbon and the Tagus Valley region pre-pubertal nine-year-old children (n = 1.496) from 2009–2013 in a descriptive, cross-sectional study. Anthropometric variables predictive power was evaluated through regression analysis.

**Results:**

At least one abnormal lipid parameter was found in 65% of “normal weight”, 73% of “overweight” and 81% of “obese” children according to the International Obesity Task Force (IOTF) standards. Dyslipidemia was present in 67.8% of children. Waist-hip ratio (WHR) explained 0.4% of total cholesterol (TC) variance. Waist circumference (WC) explained 2.8% of apolipoprotein (APO) A1 variance. Waist-circumference-to-height-ratio (WHtR) explained 2.7%, 2.8% and 1.9% of low-density lipoprotein cholesterol (LDL-c), APO B, and N_HDL-c variance, respectively. Children with abnormally high WHR levels had an increase in risk of 4.49, 3.40 and 5.30 times, respectively, for developing cardiovascular disease risk factors measured as high-risk levels of TC, LDL-c and non-HDL-c (N_HDL-c) (p<0.05). Only 29.9% of “normal weight” children had no anthropometric, bioimpedance or biochemical parameters associated with CV risk.

**Conclusion:**

A large proportion of school age children have at least one lipid profile abnormality. BMI, zBMI, calf circumference (CC), hip circumference (HC), WC, and WHR are directly associated with dyslipidemia, whereas HC and calf circumference (CC) adjusted to WC, and mid-upper arm circumference (MUAC), are all inversely associated with dyslipidemia. Selected anthropometric variables are likely to help predict increased odds of having CV risk factors.

## Introduction

Cardiovascular disease (CVD) is the major cause of morbidity and death in Western Societies [1]. CVD-related symptoms usually appear in the fourth decade of life: however, pathophysiologic process of atherosclerosis begins much earlier and is related to dyslipidemia [2]. Autopsy studies reveal that atherosclerosis lesions of fatty streak and fibrous plaques can be found as early as two years of age, and that plaque thickness is directly related to age, body mass index (BMI), total cholesterol (TC), triglycerides (TG), and low-density lipoprotein (LDL-c) levels and is inversely proportional to high-density lipoprotein (HDL-c) concentrations [3]. Because oxidative stress is increased in subclinical atherosclerosis, novel biomarkers, such as oxidized low-density lipoproteins (oxLDL), are also directly related to atherosclerosis and considered better predictors of acute coronary artery disease than standard lipid parameters or other conventional risk factors [4,5]. In adults, circulating oxLDL was shown to be associated with obesity [6], insulin resistance [7], type 2 diabetes mellitus [8], metabolic syndrome [9], and CVD [10]; whereas in children and adolescents it was associated with obesity and insulin resistance [11–13]. Previous research emphasized the contribution of excess dietary intake of cholesterol, saturated and trans fats, and carbohydrates, to the obesity epidemic and to the rising prevalence of the metabolic/insulin resistance syndrome [14]. The growing prevalence of overweight and obesity–the proportion of overweight and obese children rose 47.1% between 1980 and 2013 [15]–is a serious public health concern worldwide since obesity is known to increase insulin resistance, facilitates the development of dyslipidemia [16,17], and is considered a key pathophysiologic process that increases the risk of premature CVD [13]. Actually, studies report that such CVD risk factors are already present in children and adolescents [18,19]. Because atherosclerosis begins in childhood, the extended latent period, between elevated risk and the actual occurrence of cardiovascular (CV) outcomes, provides a window of time to explore early processes that contribute to the development of CVD and to target intervention [12]. Therefore, to slow the development of atherosclerosis and decrease CVD-related death and morbidity in adulthood, obesity and dyslipidemia prevention should begin in childhood and adolescence [3,20]. While assessment of dyslipidemia status is diagnosed through a lipid profile blood test, there is an urgent call for new potential methods to evaluate dyslipidemia risk in children that can be applied widely, such as the non-invasive anthropometric evaluation. Because children grow continuously, assessment of childhood obesity (adiposity) requires that the BMI be adjusted for sex and age (using grow charts). This anthropometric index, together with other anthropometric measures, may have unexplored potential for screening CVD-risk associated features. For instance, in a previous study [21], we developed a new sex-specific model that predicts percent body fat (% BF), as defined by bioimpedance, from simple anthropometric measurements. The aims of this study were: 1) to assess the metabolic profile of a pediatric population at a specific age; 2) to describe the association (positive or negative direction and magnitude) between anthropometric and biochemical cardiovascular disease risk factors in school-aged children; and 3) to evaluate selected anthropometric variables as potential predictors of a dyslipidemic cardiovascular risk, for the future development of an early prognostic model of cardiovascular disease risk including, although not exclusively, anthropometric measurements.

## Methods

### Study design and participants

The discriptive and cross-sectional study here reported is part of a larger project entitled "Nutritional, Biochemical and Genetic Study of an overweight and obese children population in the Southern Region". This project was approved by the Directorate General of Health, the Ministry of Science and Education of Portugal, and by the Ethics Committee of the Hospital Garcia de Orta, according to the principles of Helsinki Declaration. The project was conducted from January 2009 to June 2013 in a population of pre-pubertal children (based on Tanner stage) recruited from 87 public schools in Lisbon and the Tagus Valley metropolitan region, which included anthropometric, bioelectrical impedance, biochemical, and genetic analysis.

To be included in the study, children should have completed nine years of age during the present school year; this inclusion criterion reduced the initial population from 5.989 to 5.577 children. The exact chronologic age in days was calculated as the date of examination minus the date of birth. Children who transferred to another school, who were missing the minimum required measurements, or whose parents abandoned participation were also excluded, leaving 5.514 eligible children.

The parents of all enrolled children were required to give informed consent. Because of lack of consent for venous blood sampling of the enrolled children, the sample size for this study here declined to 3.084. Additional children were excluded if they reported to be non-fasting at the time of blood withdrawal or if their parents withdrew the consent. These exclusions produced a final analytical sample of 1.496 children. To issue the possibility of self-selection bias we compared anthropometric data between participants and non-participants. No significant differences were found (data not shown).

### Anthropometric and bioelectrical impedance analysis

Clinical procedures were performed in the schools under the supervision of two pediatric consultants. All anthropometric measurements [weight, height, BMI, BMI z-score (zBMI), waist circumference (WC), hip circumference (HC), waist-hip ratio (WHR), waist-circumference-to-height-ratio (WHtR), mid-upper arm circumference (MUAC), calf circumference (CC), bioelectrical impedance (% BF), percent skeletal muscle (% SM) and and resting metabolic rate (RMR)] were obtained with participants dressed in lightweight clothing and without shoes, using the methods previously described [21]. Children were categorized as “normal weight”, “overweight,” or “obese” according to World Obesity/Policy & Prevention (International Obesity Task Force–IOTF) cut-off values [22]. The cut-off values used to assess WC [23], WHtR [24], and % BF [25] were based on those proposed by McCarthy et al. For WHR an internal cut-off value was used (data not shown).

### Biochemical analysis

Participants were instructed to fast for 12 hours, and whole blood was sampled by venipuncture in the morning at school. Blood samples were refrigerated at approximately 5°C until transferred to our center, immediately processed for serum separation, serum aliquots frozen (-80°C) on the same day, and stored until further analysis.

Serum samples were assessed for biochemical parameters, including the characterization of the lipid profile by measurement of lipid fractions and lipoprotein: TC, HDL-c, LDL-c, non-HDL cholesterol (N_HDL-c) and triglycerides (TG), as well as apolipoproteins A1 (Apo A1) and B (Apo B), using turbidimetric immuno-enzymatic assays. Dyslipidemia was defined as the presence of one or more parameters of the serum lipid profile beyond the normal range. Abnormal lipid concentrations in blood were defined by established cut-offs as follows: total cholesterol >170 mg/dl, LDL-c >110 mg/dl, and N_HDL-c ≥120 mg/dl, as defined by the American Academy of Pediatrics, [26] and HDL-c <40 mg/dl, triglycerides > 75 mg/dl, APO A1 <1.2 g/L, and APO B > 0.9 g/L, as defined by the Laboratories of the Mayo Clinic [27].

Serum samples were also tested for glucose (Glucose oxidase method), creatinine (Jaffé method), total proteins (Biuret method), and ferritin (immunoassay method); normal values were defined as follows: glucose <100 mg/dl, creatinine 0.4–1.0 mg/dl, total proteins 6–8.3 mg/dl, and ferritin 7–140 ng/ml [26].

All biochemical determinations were performed using *Horiba Medical* reagents in a *Horiba Pentra C400* auto analyzer (France), and obtained inter and intra-assay coefficients of variation were always <3%.

Insulin (kit—10-1113-01, Mercodia), leptin (kit– 10-1199-01, Mercodia), and oxLDL (kit– 10-1143-01, Mercodia) were measured by ELISA in a DS2 auto analyzer from *Dynex Technologies™* (Magellan Biosciences, USA). The homeostasis model assessment of insulin resistance (Homa-IR), was calculated from glucose (mg/dl) and insulin (μU/ml), using the following formula: Homa-IR = (insulin (μU/ml) x glucose (mg/dl))/405. For these parameters internal cut-off values were used. Normal values were defined as follows: insulin < 16.3μU/ml, Homa-IR < 0.69, leptin < 6.32 ng/ml, and oxLDL < 1.38mU/L.

### Statistical analysis

The statistical analysis was performed with SPSS for Windows statistical package (SPSS Inc., Chicago, version 21). Descriptive statistics for anthropometric and biochemical/clinical parameters were calculated by IOTF categories, ethnicity groups and gender, and differences between means were t-tested with Bonferroni adjustment. The relationships between anthropometric and biochemical variables were evaluated through partial correlation coefficients, stepwise (bidirectional) multiple linear regression and stepwise backwards (Wald) logistic regression. Both regression analyses were fitted including the constant and hierarchically adjusted for age, gender, and ethnicity with selected coefficients internally validated by simple bootstrap (1000 samples) with bias corrected and accelerated 95% confidence interval. Girls were the reference attribute (value = 0) for Gender. Caucasians were the reference attribute (value = 0) for ethnicity. The stepwise linear regression used the F-statistic p-value as criterion for independent variable insertion (0.05) or removal (0.10). The dependent variables were pediatric biochemical/clinical parameters (lipid profile and oxLDL), whereas the independent variables (covariates) were the anthropometric parameters (BMI, zBMI, WC, HC, WHR, WHtR, MUAC, and CC). The influence of selected anthropometric variables on the variance of the assumed biochemical cardiovascular risk factors was evaluated through the cumulative adjusted r^2^. Effect sizes of selected independent variables were estimated by the models standardized coefficients (Beta). Next, we used IOTF criteria for normal weight in combination with established biochemical and anthropometric pediatric cut-off points as criteria to select an “apparently healthy” subset of children from the study population, and thereafter define internal cut-off values for those variables without known pediatric cut-off values as the higher value for each in this subset. These cut-offs were used to turn the dependent and independent continuous variables into binomial ones, as each variable value was classified with the normal (0) or high value (1, risk factor) categorical attribute. Odds ratios were calculated using as dependent variables the binomial biochemical/clinical risk factor, and as independent binomial variables the anthropometric parameters. Cases with missing data were not included in the analysis. Significance was considered as p-value <0.05 for all tests.

## Results

We characterized a cohort of 1,496 schoolchildren (723 boys and 773 girls) with a mean age of 9.75 years according to anthropometric, bioimpedance and biochemical parameters. According to IOTF criteria, 71.5% were "normal weight" and 28.5% were "overweight", including 7.6% who were "obese". Racial-ethnic composition was 87.5% Caucasian, 11.4% Afro-Portuguese, and 1.1% other ethnicities. Caucasian and Afro-Portuguese children differed in all anthropometric and bioimpedance parameters with the exception of height and % SM; Caucasians had higher values (p<0.05) (S1 Table). No significant differences in biochemical levels were found between the two groups (p>0.05), except for total proteins (S1 Table). For this reason, these two ethnic groups were merged in subsequent analyses. Other race-ethnic groups were not included; the sample sizes of the other groups were too small to justify a separate analysis. No differences were found between boys and girls in anthropometric and bioimpedance parameters with the exception of height, WHR, % SM and RMR (p >0.05) (Table 1). In contrast, significant differences were observed between genders for glycemia, lipid profile (except for TC and LDL-c), total protein, creatinine, and leptin levels; girls had higher mean values, with the exception of glycemia, HDL-c, APO A1, and creatinine (p <0.05) (Table 1).

**Table 1:** Descriptive characteristics of the study population of children by gender.

*Characteristic*	Total (n = 1496)	Male (n = 723)	Female (n = 773)
	Mean ± SD	Mean ± SD	Mean ± SD
Age^§^	9.74 ± 0.61	9.76a ± 063	9.71a ± 0.58
***Anthropometry***			
Weight (Kg)	35.74 ± 8.72	35.98_a_ ± 8.92	35.52_a_ ± 8.52
Height (cm)	138.5 ± 7.4	138.9_b_ ± 7.3	138.1_a_ ± 7.5
BMI (Kg/m^2^)	18.44 ± 3.24	18.44_a_ ± 3.29	18.44_a_ ± 3.18
zBMI	0.67 ± 1.08	0.67_a_ ± 1.1	0.67_a_ ± 1.07
WC (cm)	65.25 ± 9.15	65.0_a_ ± 9.1	65.5_a_ ± 9.2
HC (cm)	71.8 ± 8	71.3_a_ ± 8.1	72.3_a_ ± 7.9
WHR (WC/HC)	0.89 ± 0.05	0.90_b_ ± 0.05	0.89_a_ ± 0.05
WHtR (WC/height)	0.472 ± 0.06	0.47_a_ ± 0.06	0.48_a_ ± 0.06
MUAC (cm)	21.2 ± 2.9	21.1_a_ ± 3	21.3_a_ ± 2.7
CC (cm)	29.2 ± 3.4	29.2_a_ ± 3.4	29.1_a_ ± 3.3
***Bioelectrical impedance***			
BF (%)	21.9 ± 7.7	21.4_b_ ± 7.5	22.4_a_ ± 7.9
SM (%)	31.9 ± 2.8	32.6_b_ ± 2.74	31.2_a_ ± 2.65
RMR (Kcal/day)	1211 ± 116	1244_b_ ± 126	1181_a_ ± 96
***Biochemical Parameters***			
Glycemia (mg/dl)	78.1 ± 12.8	79.1_b_ ± 11.8	76.8_a_ ± 9.7
TC (mg/dl)	170.6 ± 29.5	169.4_a_ ± 29.4	171.6_a_ ± 29.5
LDL-c (mg/dl)	93.8 ± 24.6	91.1_b_ ± 24.3	96.2_a_ ± 24.6
HDL-c (mg/dl)	56.5 ± 11.2	57.5_b_ ± 11.5	55.6_a_ ± 10.9
TG (mg/dl)	62.3 ± 26.8	58.8_b_ ± 26.4	65.2_a_ ± 26.1
APO A1 (g/L)	1.36 ± 0.19	1.38_b_ ± 0.19	1.34_a_ ± 0.18
APO B (g/L)	0.74 ± 0.17	0.72_b_ ± 0.17	0.76_a_ ± 0.16
APO B/APO A1	0.6 ± 0.1	0.5_b_ ± 0.1	0.6_a_ ± 0.1
LDL-c/Apo B	1.27 ± 15.1	1.27_a_ ± 1.6	1.26_a_ ± 1.4
TC/HDL	3.09 ± 0.63	3.02_b_ ± 0.63	3.16_a_ ± 0.61
N_ HDL-c (mg/dl)	114 ± 26.7	111_b_ ± 26.7	116_a_ ± 26.4
LDL/HDL	1.72 ± 0.56	1.65_b_ ± 0.55	1.79_a_ ± 0.55
Total Proteins (mg/dl)	7.3 ± 0.7	7.2_b_ ± 0.67	7.4_a_ ± 0.73
Ferritin (ng/ml)	38.8 ± 21.3	38.9_a_ ± 20.3	38.7_a_ ± 22.2
Creatinine (mg/dl)	0.6 ± 0.11	0.61_b_ ± 0.09	0.59_a_ ± 0.11
Insulin (μU/ml)^1^	7.2 ± 11.19	6.81_a_ ± 11.74	7.45_a_ ± 10.59
Homa-IR	1.41 ± 2.47	1.26_a_ ± 1.97	1.53_a_ ± 2.83
Leptin (ng/ml) ^2^	10.71 ± 10.8	8.73_b_ ± 9.83	12.56_a_ ± 11.7
oxLDL (mU/L) ^3^	6.55 ± 1.78	6.34_a_ ± 1.71	6.75_a_ ± 1.82

^§^Age in days were converted to years for between-group comparisons.

^1^(Female-272/Male-246)

^2^ (Female -266/Male -246)

^3^ (Female F-129/Male -133).

Gender means of each characteristic are compared through Bonferroni-adjusted t-tests, and statistical significance of differences are reported as different associated letters: a.b) (p<0.05). APO A1 (apolipoprotein A1); APO B (apolipoprotein B); BMI (body mass index); CC (calf circumference); HC (hip circumference); HDL-c (high-density lipoproteins cholesterol); LDL-c (low-density lipoproteins cholesterol); HOMA-IR (homeostatic model assessment-insulin resistance); MUAC (mid-upper arm circumference); N_HDL-c (non-HDL cholesterol); oxLDL (oxidized low-density lipoprotein); RMR (resting metabolic rate); TC (total cholesterol); TG (triglycerides); WC (waist circumference); WHR (waist-hip ratio); WHtR (waist circumference-to-height-ratio); zBMI (BMI z-score); % BF (percent body fat); and % SM (percent skeletal muscle).

Significant differences in all anthropometric and bioimpedance measurements were observed between children categorized to the “normal weight”, “overweight” and “obese” groups (p <0.05) (S2 Table). No significant differences were observed in glucose, TC, total proteins and creatinine levels between the weight groups. However, the weight groups differed in HDL-c, TG, APO A1, TC/HDL, LDL/HDL, and leptin mean values (p<0.05) (Table 2). In contrast to other biochemical parameters, HDL-c and Apo A1 were significantly lower in the "overweight" and "obese" groups. “Normal weight” and “overweight/obese” groups differed significantly in LDL-c, APO B, APO B/APO A1, N_HDL-c, ferritin, insulin, Homa-IR, and oxLDL mean values. “Normal and overweight” children had significantly lower LDL/APO B than those within the “obese” category. Differences found between boys and girls of the “normal weight” group were the same as those previously described for the overall population, whereas no differences other than in leptin levels were found when “overweight” and “obese” gender groups were compared. “Overweight” and “obese” boys also had higher levels of creatinine and APO A1, respectively.

**Table 2:** Descriptive biochemical characteristics by gender and IOTF category^Δ^.

*Biochemical parameters*	Normal			Overweight			Obese		
	Total (n = 1071)	Male (n = 523)	Female (n = 548)	Total (n = 312)	Male (n = 140)	Female (n = 172)	Total (n = 113)	Male (n = 60)	Female (n = 53)
	Mean ± SD	Mean ± SD	Mean ± SD	Mean ± SD	Mean ± SD	Mean ± SD	Mean ± SD	Mean ± SD	Mean ± SD
Glucose (mg/dl)	77.7 ± 13.1*	78.6_b_ ± 10.8	76.2_a_ ± 9.4	79.4 ± 12.6*	80.9_a_ ± 15.3	78.2_a_ ± 9.9	78.9±10.8*	79.1_a_±10.6	78.6_a_ ± 11.2
TC (mg/dl)	169.9 ± 29.0*	167.6_b_ ± 28.0	171.9_a_ ± 29.7	172.5 ±30.3*	173.3_a_ ± 30.7	171.8_a_ ±30.1	172.3±31.5*	175.7_a_±36.5	168.6_a_ ±24.6
LDL-c (mg/dl)	91.7 ± 23.7*	87.7_b_ ± 22.4	95.1_a_ ± 24.3	98.5 ±25.3**	98.1_a_ ± 24.7	98.8_a_ ± 25.9	101.0±27.5**	103.6_a_ ±31.2	98.2_a_ ± 22.6
HDL-c (mg/dl)	57.9 ± 11.3*	59.2_b_ ± 11.5	56.8_a_ ± 11.1	54.2 ± 10.3*	54.6_a_ ± 10.4	53.9_a_ ± 10.3	49.0 ± 8.1***	49.6_a_ ± 8.9	48.4_a_ ± 7.3
TG (mg/dl)	58.0 ± 23.1*	53.6_b_ ± 20.8	61.8_a_ ±23.2	68.2 ±28.8**	66.0_a_ ± 30.9	70.0_a_ ±26.9	86.2 ± 36.5***	87.3_a_ ± 35.2	84.9_a_ ± 38.2
APO A1 (g/L)	1.37 ± 0.2*	1.39_b_ ± 0.2	1.35_a_ ± 0.2	1.33 ± 0.2**	1.35_a_ ± 0.2	1.32_a_ ± 0.2	1.27 ± 0.2***	1.31_b_ ± 0.2	1.24_a_ ± 0.1
APO B (g/L)	0.72 ± 0.2*	0.70_b_ ± 0.2	0.75_a_ ± 0.2	0.77 ± 0.2**	0.76_a_ ± 0.2	0.78_a_ ± 0.2	0.78 ± 0.2**	0.80_a_ ± 0.2	0.76_a_ ± 0.2
APO B/APO A1	0.5 ± 0.1*	0.5_b_ ± 0.1	0.6_a_ ± 0.1	0.6 ± 0.1**	0.6_a_ ± 0.1	0.6_a_ ± 0.1	0.6 ± 0.2**	0.6_a_ ± 0.2	0.6_a_ ± 0.2
LDL-c/Apo B	1.26 ± 1.6*	1.26_a_ ± 1.7	1.26_a_ ± 1.4	1.27 ± 1.5*	1.29_a_ ± 1.5	1.27_a_ ± 1.4	1.29 ± 1.2**	1.29_a_ ± 1.1	1.29_a_ ± 1.4
TC/HDL	3.0 ± 0.6*	2.9_b_ ± 0.5	3.1_a_ ± 0.6	3.25 ± 0.6**	3.2_a_ ± 0.7	3.3_a_ ± 0.6	0.6 ± 0.8***	3.6_a_ ± 0.9	3.6_a_ ± 0.7
N_ HDL-c (mg/dl)	112.0 ±25.7*	108.4_b_ ± 24.5	115.1_a_ ± 26.2	118.3±27.7**	118.7_a_ ±27.8	118.0_a_ ±27.7	122.7 ± 30.0**	124.9_a_ ±34.4	120.2_a_±24.4
LDL/HDL	1.64 ± 0.5*	1.54_b_ ± 0.5	1.73_a_ ± 0.5	1.87 ± 0.6**	1.85_a_ ±0.6	1.89_a_ ±0.6	2.1 ± 0.7***	2.13_a_ ± 0.7	2.08_a_ ± 0.6
Total Proteins (mg/dl)	7.26 ± 0.7*	7.19_b_ ± 0.6	7.34_a_ ± 0.7	7.40 ± 0.8*	7.35_a_ ±0.8	7.43_a_ ±0.9	7.40 ± 0.7*	7.34_a_ ± 0.8	7.45_a_ ± 0.6
Ferritin (ng/ml)	37.3 ± 19.7*	37.1_a_ ± 17.7	37.5_a_ ± 21.5	41.4 ± 24.6**	43.6_a_ ±27.5	39.6_a_ ±21.8	45.5 ± 23.9**	42.9_a_ ± 19.6	48.4_a_±27.7
Creatinine (mg/dl)	0.6 ± 0.1*	0.6_b_ ± 0.1	0.59_a_ ± 0.1	0.6 ± 0.1*	0.62_b_ ±0.1	0.58_a_ ±0.1	0.6 ± 0.1*	0.59_a_ ± 0.1	0.62_a_ ± 0.1
Insulin (μU/ml)^1^	5.09 ± 4.2*	4.8_a_ ± 4.2	5.2_a_ ± 3.5	9.91 ± 16.2**	10.3_a_ ± 20.1	9.6_a_ ± 12.4	1.2 ± 16.9**	8.6_a_ ± 9.8	14.3_a_±22.49
Homa-IR	0.96 ± 0.9*	0.93_a_ ± 0.8	0.97_a_ ± 0.8	1.88 ± 3.0**	1.72_a_ ± 3.2	2.0_a_ ± 2.9	2.47 ± 4.9**	1.80_a_ ± 2.3	3.26_a_ ± 6.8
Leptin (ng/ml)^2^	5.7 ± 6.3*	3.9_b_ ± 1.6	6.7_a_ ± 7.1	14.7 ± 6.4**	12.0_a_ ± 6.7	17.1_b_ ± 7.4	27.1 ± 16.5***	24.3_b_ ± 13.5	31.8_a_±12.6
oxLDL (mU/L)^3^	6.33 ± 1.6*	6.2_a_ ± 1.6	6.5_a_ ± 1.6	7.0 ± 1.9**	6.9_a_ ± 1.8	7.1_a_ ± 1.9	7.0 ± 2.3**	6.6_a_ ± 2.0	7.3_a_ ± 2.6

^Δ^According to World Obesity/Policy & Prevention analysis cut-off values. Gender means of each characteristic were compared within each IOTF category whereas IOTF Grade means of each characteristic were compared through Bonferroni-adjusted t-tests. Statistical differences were reported as different associated letters (a.b) or symbols (*.**.***), respectively (p < 0.05).

^1^(Female-272/Male-246)

^2^(Female-266/Male-246)

^3^(Female-129/Male-133).

APO A1 (apolipoprotein A1): APO B (apolipoprotein B): HDL-c (high-density lipoproteins cholesterol): LDL-c (low-density lipoproteins cholesterol): HOMA-IR (homeostatic model assessment-insulin resistance): N_HDL-c (non-HDL cholesterol): oxLDL (oxidized low-density lipoprotein): TC (total cholesterol): and TG (triglycerides).

Correlation analysis of lipid profile parameters showed that anthropometric parameters (except for WHtR and HC for LDL/HDL) were positively and moderately correlated with TG, TC/HDL and LDL/HDL and inversely correlated to HDL-c (p <0.05) (Table 3). Apo B/APO A1 was moderately and positively correlated with BMI and WHR (p <0.05).

**Table 3:** Correlations between anthropometric and biochemical characteristics adjusted for age, gender and ethnicity.

	Glucose (mg/dl)	Insulin (mU/L)	Homa-IR	TC (mg/dl)	LDL-c (mg/dl)	HDL-c (mg/dl)	TG (mg/dl)	APO A1 (g/L)	APO B (g/L)	APO B /APO A1	LDL-c/ Apo B	TC/ HDL	N_HDL-c (mg/dl)	LDL/ HDL	Leptin (ng/ml)	oxLDL (mU/L)
Weight (Kg)	0.227	0.294	0.270	NS	0.074	-0.271	0.303	-0.193	0.075	0.16	0.03	0.245	0.066	0.232	0.774	NS
Height (cm)	NS	0.228	0.211	NS	NS	NS	NS	NS	NS	NS	NS	NS	NS	NS	0.297	NS
BMI (Kg/m^2^)	0.238	0.217	0.196	NS	0.147	-0.263	0.318	-0.175	0.143	0.201	0.066	0.287	0.128	0.282	0.856	0.159
zBMI	0.254	0.213	0.19	NS	0.138	-0.25	0.267	-0.173	0.136	0.197	0.058	0.264	0.116	0.263	0.771	0.143
WC (cm)	0.141	0.185	0.167	NS	0.115	-0.253	0.264	-0.174	0.119	0.19	N/S	0.257	0.098	0.254	0.877	NS
WHR (WC/HC)	0.125	0.104	0.091	NS	0.168	-0.224	0.249	-0.144	0.168	0.211	0.068	0.271	0.142	0.276	0.879	NS
HC (cm)	0.149	0.247	0.219	NS	N/S	-0.24	0.246	-0.16	0.075	0.145	N/S	0.202	N/S	0.196	0.814	NS
WHtR (WC/height)	0.015	-0.057	-0.045	0.072	0.134	-0.096	0.112	-0.074	0.118	0.14	0.086	0.177	0.12	0.183	0.652	0.145
MUAC (cm)	0.212	0.191	0.165	NS	0.107	-0.238	0.233	-0.165	0.092	0.161	N/S	0.217	0.07	0.231	0.802	0.135
CC (cm)	NS	NS	NS	NS	NS	NS	NS	NS	NS	NS	NS	NS	NS	NS	0.527	NS

Correlation coefficients are displayed if statistically significant P <0.05. NS (not significant): APO A1 (apolipoprotein A1): APO B (apolipoprotein B): BMI (body mass index): CC (calf circumference): HC (hip circumference): HDL-c (high-density lipoproteins cholesterol): LDL-c (low-density lipoproteins cholesterol): HOMA-IR (homeostatic model assessment-insulin resistance): MUAC (mid-upper arm circumference): N_HDL-c (non-HDL cholesterol): oxLDL (oxidized low-density lipoprotein): TC (total cholesterol): TG (triglycerides): WC (waist circumference): WHR (waist-hip ratio): WHtR (waist circumference-to-height-ratio): and zBMI (BMI z-score).

BMI (r = 0.159), zBMI (r = 0.143), WHtR (r = 0.145), and MUAC (r = 0.135) were positively correlated with oxLDL (p<0.05). Other parameters, such as glucose and insulin, were moderately and significantly correlated with weight, BMI and zBMI (p <0.05). Glucose was also moderately correlated with MUAC and insulin with HC (p <0.05). We found the strongest correlations between leptin and all anthropometric parameters (p <0.05). The correlation analysis of biochemical variables showed that oxLDL was strongly and significantly associated with TC, LDL-c, APO B, APO B/APO A1, TC/HDL, and N_HDL-c (S3 Table).

A careful analysis of these previous results showed a significant percentage of children within normal IOTF values had anthropometric, bioimpedance or biochemical values outside the pediatric reference range. Among 1.071 “normal weight” children only 320 (29.9%) had anthropometric, % BF, and biochemical parameters all within the normal range (Table 4).

**Table 4:** Descriptive characteristics of the subset of children without risk factors by gender.

*Characteristic*	Total (n = 320)	Male (n = 174)	Female (n = 146)
	Mean ± SD	Mean ± SD	Mean ± SD
Age^§^	9.77 ± 0.57	9.7_a_ ± 0.57	9.8_a_ ± 0.57
***Anthropometry***			
Weight (Kg)	31.5 ± 5.1	31.64_a_ ± 4.71	31.3_a_ ± 5.5
Height (cm)	137.3 ± 7.2	137.5_a_ ± 7.0	137.1_a_ ± 7.6
BMI (Kg/m^2^)	16.6 ± 1.6	16.7_a_ ± 1.5	16.5_a_ ± 1.7
zBMI	0.06 ± 0.74	0.1_a_ ± 0.7	0.1_a_ ± 0.78
WC (cm)	60.6 ± 5.3	60.4_a_ ± 4.7	60.7_a_ ± 5.9
HC (cm)	68.1 ± 5.7	67.3_b_ ± 5.2	69.2a ± 6.1
WHR (WC/HC)	0.88 ± 0.05	0.89_b_ ± 0.04	0.87_a_ ± 0.05
WHtR (WC/height)	0.44 ± 0.03	0.44_a_ ± 0.03	0.44_a_ ± 0.04
MUAC (cm)	19.8 ± 1.9	19.7_b_ ± 1.9	19.9_a_ ± 2.0
CC (cm)	27.6 ± 2.3	27.7a ± 2.2	27.5_a_ ± 2.5
***Bioelectrical impedance***			
BF (%)	17.7 ± 5.5	17.5_a_ ± 5.3	17.9_a_ ± 5.7
SM (%)	32.5 ± 3.22	33.1_b_ ± 3.1	31.7_a_ ± 3.2
RMR (Kcal/Day)	1168 ± 85	1193_b_ ± 84	1138_a_ ± 76
***Biochemical Parameters***			
Glucose (mg/dl)	76.1 ± 9.3	76.4_a_ ± 9.5	75.8_a_ ± 9.0
TC (mg/dl)	151.3 ± 13.7	150.5_a_ ± 13.6	152.3_a_ ± 13.7
LDL-c (mg/dl)	76.6 ± 15.3	74.7_b_ ± 16.0	78.8_a_ ± 14.2
HDL-c (mg/dl)	58.3 ± 8.0	59.6_b_ ± 8.5	56.9_a_ ± 7.2
TG (mg/dl)	47.4 ± 12.5	45.7_b_ ± 12.5	49.4_a_ ± 12.3
APO A1 (g/L)	1.39 ± 0.12	1.40_a_ ± 0.13	1.37_a_ ± 0.12
APO B (g/L)	0.62 ± 0.1	0.60_b_ ± 0.1	0.65_a_ ± 0.1
APO B/APO A1	0.5 ± 0.1	0.4_b_ ± 0.1	0.5_a_ ± 0.1
LDL-c/Apo B	1.22 ± 12.0	1.23_a_ ± 12.6	1.22_a_ ± 11.7
TC/HDL	1.4 ± 0.4	1.3_b_ ± 0.4	1.4_a_ ± 0.33
N_ HDL-c (mg/dl)	93.0 ± 14.2	91.0_b_ ± 14.3	95.5_a_ ± 13.63
LDL/HDL	2.6 ± 0.4	2.6_b_ ± 0.4	2.7_a_ ± 0.36
Total Proteins (mg/dl)	7.1 ± 0.46	7.1_a_ ± 0.46	7.1_a_ ± 0.46
Ferritin (ng/ml)	35.8 ± 16.2	35.3_a_ ± 15.3	36.3_a_ ± 17.4
Creatinine (mg/dl)	0.60 ± 0.08	0.61_b_ ± 0.09	0.58_a_ ± 0.08
Insulin (μU/ml)^1^	4.6 ± 3.2	4.4_a_ ± 2.7	4.8_a_ ± 3.7
Homa-IR	0.89 ± 0.69	0.84_a_ ± 0.61	0.93_a_ ± 0.77
Leptin (ng/ml)^2^	5.7 ± 6.3	5.1_a_ ± 7.5	6.3_a_ ± 5.1
oxLDL (mU/L)^3^	5.8 ± 1.4	5.7_a_ ± 1.3	5.9_a_ ± 1.48

^§^Age in days are converted in years for a better comparison between groups.

^1^ (Female-38/ Male-37)

^2^ (Female-40/Male-36)

^3^ (Female-24/Male-31).

Gender means are compared between each characteristic through Bonferroni-adjusted t-tests and statistical differences are reported as different associated letters: a.b (p < 0.05). Values outside the following biochemical normal cut-offs were considered risk factors: glucose < 100mg/dl. TC < 170 mg/dl. LDL-c < 110 mg/dl. HDL-c ≥ 40 mg/dl. TG ≤ 75 mg/dl. APO A1 > 1.2 g/L. APO B < 0.9 g/L. N_HDL-c < 120. Total proteins 6–8.3 mg/dl. Ferritin 7–140 ng/ml. creatinine 0.4–1.0 mg/dl. Insulin < 16.3 μU/ml. Homa-IR < 0.69. Leptin < 6.32 ng/ml and ox-LDL < 1.38 mU/L. APO A1 (apolipoprotein A1). APO B (apolipoprotein B): BMI (body mass index): CC (calf circumference): HC (hip circumference): HDL-c (high-density lipoproteins cholesterol): LDL-c (low-density lipoproteins cholesterol): HOMA-IR (homeostatic model assessment-insulin resistance): MUAC (mid-upper arm circumference): N_HDL-c (non-HDL cholesterol): oxLDL (oxidized low-density lipoprotein): RMR (resting metabolic rate): TC (total cholesterol): TG (triglycerides): WC (waist circumference): WHR (waist-hip ratio): WHtR (waist circumference-to-height-ratio): zBMI (BMI z-score): % BF (percent body fat): and % SM (percent skeletal muscle).

**Fig 1:**
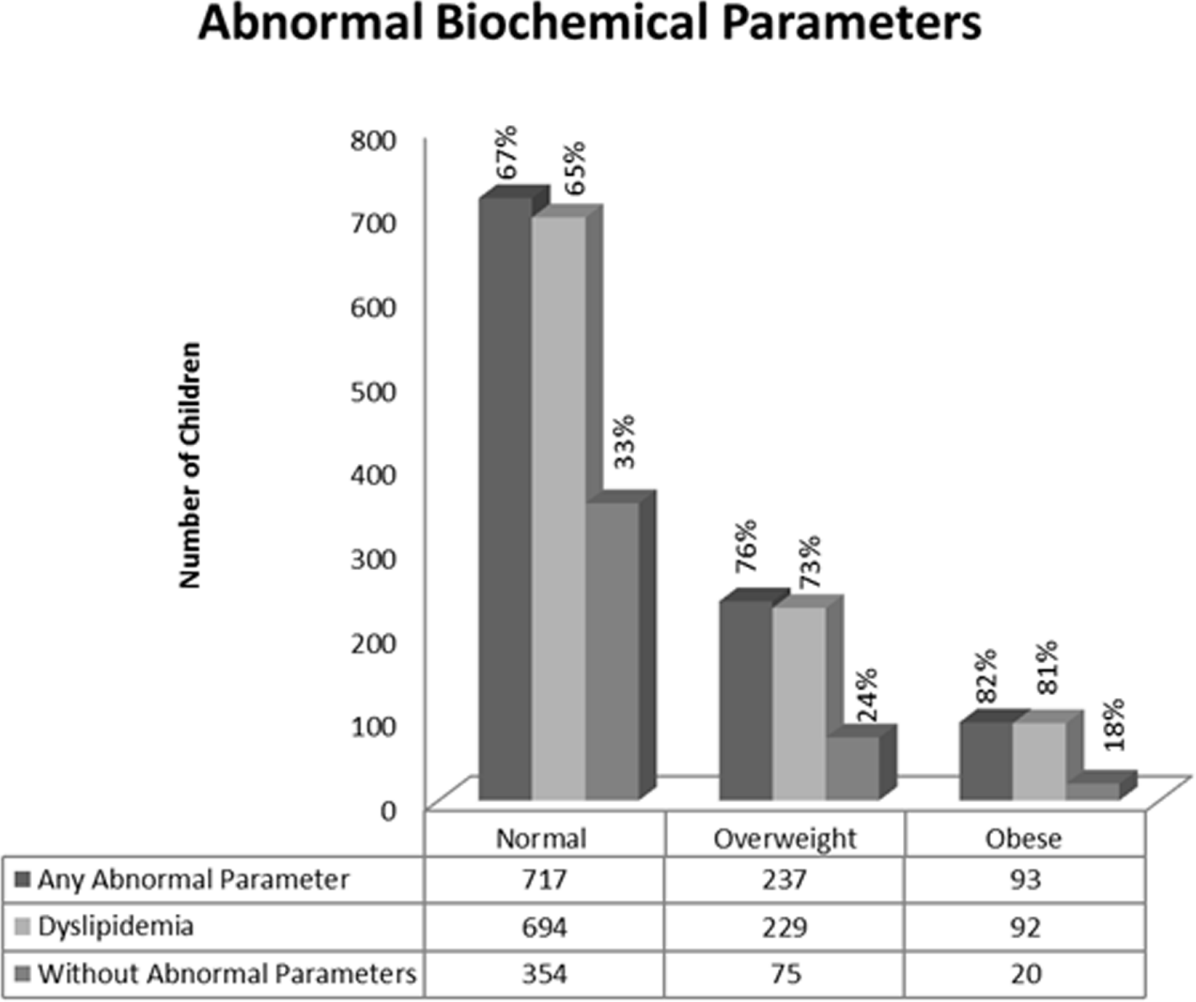
Distribution of abnormal biochemical parameters according to IOTF category.

The observation of abnormal parameters within the normal IOTF group led us to question how many children would meet all the pediatric reference criteria. The number of children with dyslipidemia and/or any other abnormal biochemical parameter is shown in Fig 1. After evaluating the number of dyslipidemic disorders, 65% of “normal weight”, 73% of “overweight” and 81% of “obese” children had at least one abnormal lipid parameter equivalent to a CV risk factor. The risk of dyslipidemia was associated with zBMI. In addition, only 33% of “normal weight”, 24% of “overweight”, and 18% of “obese” children lacked any abnormal biochemical parameter among the parameters measured.

Results of the multiple linear regression analyses for lipid profile measures are presented in Table 5. WHR, WHtR, WC, HC, BMI and zBMI were significant determinants of lipid and lipoproteins levels depending on the analyzed biochemical parameter. However, the percentage of variance of lipid levels explained by these parameters was very low. CC and MUAC, after adjustment for WC, were inversely associated with high-risk levels of TG and HDL-c, indicating a protective effect against dyslipidemia (S4 Table). Results in Table 5 also show that WHR was a significant predictor for TC and N_HDL-c; it equally explained 0.4% of TC and N_HDL-c variances. WC explained 2.8%, 1.2%, and 0.8% of APO A1, APO B and N_HDL-c variance, respectively. WHtR explained 2.7%, 2.8%, and 1.9% of LDL-c, APO B, and N_HDL-c variance, respectively. BMI explained 0.4%, 9.7% and 0.3% of LDL-c, TG and N_HDL-c, respectively. Finally, HC explained 0.3% of LDL-c and zBMI explained 2.4% of TG. Male gender consistently showed a protective effect towards dyslipidemia. WHtR and BMI showed a positive (increasing) effect over LDL-c and N_HDL-c, favouring dyslipidemia in general.

**Table 5:** Final multiple regression models that explain the variance of blood lipids and lipoproteins. Independent control variables were gender, age and ethnicity; anthropometric measurements were independent covariates.

Dependent Variable (n = 1496)	Independent Variable	Std. Coef.	Coef. Sig.	Cumulative Adjusted r^2^	Coef. Bootstrap Sig.
TC	Constant		0.00		<0.05
	Gender*	-0.07	0.02		<0.05
	Age	-0.04	0.22		0.21
	Ethnicity	0.04	0.20	0.004	0.24
	WHR*	0.07	0.03	0.008	<0.05
LDL-c	Constant		0.02		<0.05
	Gender*	-0.12	0.00		<0.05
	Age	0.07	0.06		0.07
	Ethnicity	0.05	0.10	0.011	0.13
	WHtR*	0.33	0.00	0.038	<0.05
	WC	-0.24	0.07	0.050	0.07
	BMI*	0.22	0.00	0.054	<0.05
	HC*	-0.18	0.03	0.057	<0.05
HDL-c	Constant		0.00		<0.05
	Gender*	0.10	0.00		<0.05
	Age*	-0.08	0.01		<0.05
	Ethnicity	0.01	0.88	0.014	0.81
	BMI*	-0.26	0.00	0.081	<0.05
TG	Constant		0.00		<0.05
	Gender*	-0.15	0.00		<0.05
	Age	0.00	0.93		0.89
	Ethnicity	0.01	0.84	0.026	0.80
	BMI*	0.92	0.00	0.123	<0.05
	zBMI*	-0.63	0.00	0.147	<0.05
APO A1	Constant		0.00		<0.05
	Gender*	0.10	0.00		<0.05
	Age*	-0.09	0.01		<0.05
	Ethnicity	-0.03	0.43	0.018	0.48
	WC*	-0.20	0.00	0.046	<0.05
APO B	Constant		0.01		<0.05
	Gender*	-0.13	0.00		<0.05
	Age*	0.07	0.04		<0.05
	Ethnicity	0.06	0.70	0.015	0.75
	WHtR*	0.46	0.00	0.043	<0.05
	WC*	-0.31	0.00	0.055	<0.05
N_HDL-c	Constant		0.10		0.09
	Gender*	-0.12	0.00		<0.05
	Age	0.04	0.23		0.24
	Ethnicity	0.05	0.11	0.010	0.10
	WHtR*	0.25	0.01	0.029	<0.05
	WC*	-0.38	0.00	0.037	<0.05
	BMI*	0.22	0.00	0.040	<0.05
	WHR*	0.10	0.02	0.044	<0.05
oxLDL	Constant		0.00		
	Gender*	-0.21	0.03		<0.05
	Age	-0.16	0.08		0.08
	Ethnicity	0.09	0.35	0.000	0.33

*Variable with significant coefficient for p<0.05.

Std. Coef. (Standardized coefficient–Beta effect size). Sig. (Coefficients Significance). Bootstrap Sig. (Significance of Coefficients estimated by bootstrap). Statistically significant P <0.05. APO A1 (apolipoprotein A1): APO B (apolipoprotein B): BMI (body mass index): CC (calf circumference): HC (hip circumference): HDL-c (high-density lipoproteins cholesterol): LDL-c (low-density lipoproteins cholesterol): MUAC (mid-upper arm circumference): N_HDL-c (non-HDL cholesterol): oxLDL (oxidized low-density lipoprotein): TC (total cholesterol): TG (triglycerides): WC (waist circumference): WHR (waist-hip ratio): WHtR (waist circumference-to-height-ratio): and zBMI (BMI z-score).

The independent contributions of anthropometric parameters to predict an abnormal lipid profile (CVD risk factor), according to logistic models adjusted for ethnicity, age, and gender, are shown in Table 6. WHR was found to be the strongest predictor of those with CVD risk levels of TC, LDL-c, and N_HDL-c. Children with abnormal WHR levels had 4.5 (95% CI: 1.4–14.0), 3.4 (95% CI: 1.3–9.1), and 5.3 (95% CI: 1.7–16.5) times more risk of having a high risk level of TC, LDL-c and N_HDL-c (p<0.05) than children with normal WHM levels, respectively. zBMI was the only independent predictor of oxLDL. In fact, children in the category of high zBMI had, on average, a 4.12 (95% CI: 1.20–14.15) times higher risk of having a high-risk level of oxLDL than those children with normal zBMI levels.

**Table 6:** Independent contributions of anthropometric parameters to the prediction of cardiovascular disease risk factors (adjusted for gender, age and ethnicity).

CVD Risk Factors (n = 1496)	Anthropometric variables	Odds ratio (95% CI)	Bootstrap Sig.	AUC (95% CI)
TC	HC	0.481 (0.206; 1.124)	0.10	
	WHR*	4.491 (1.435; 14.054)	<0.05	0.66 (0.52; 0.59)
LDL-c	BMI*	1.733 (1.26; 2.38)	<0.05	
	WHR*	3.40 (1.27; 9.10)	<0.05	0.61 (0.57; 0.65)
HDL-c	WC*	4.69 (1.78; 12.40)	<0.05	0.64 (0.56; 0.72)
TG	BMI*	2.21 (1.52; 3.23)	<0.05	
	WHtR	1.65 (0.95; 2.87)	0.09	
	CC	1.63 (0.98; 2.72)	0.07	0.66 (0.62; 0.70)
Apo A1	HC*	4.17 (1.44; 12.06)	<0.05	
	CC*	2.29 (1.24; 4.23)	<0.05	
	MUAC*	0.17 (0.04; 0.77)	<0.05	0.64 (0.59; 0.68)
Apo B	zBMI*	2.10 (1.43; 3.08)	<0.05	0.64 (0.60; 0.68)
N_HDL-c	BMI*	1.53 (1.13; 2.06)	<0.05	
	WHR*	5.30 (1.70; 16.48)	<0.05	0.59 (0.56; 0.63)

*Variable with significant coefficient for p<0.05.

Bootstrap Sig. (Significance of Coefficients estimated by bootstrap). AUC (area under the curve). CVD (cardio-vascular disease). CI (confidence interval). CV high risk lipid concentrations, based on established cut-offs values, were defined as follows: total cholesterol >170 mg/dl; LDL-c >110 mg/dl; HDL-c <40 mg/dl; triglycerides >75 mg/dl; APO A1 <1.2 g/L; APO B >0.9 g/L; N_HDL-c ≥120 mg/dl, and ox-LDL >1.38 mU/L. APO A1 (apolipoprotein A1): APO B (apolipoprotein B): BMI (body mass index): CC (calf circumference): HC (hip circumference): HDL-c (high-density lipoproteins cholesterol): LDL-c (low-density lipoproteins cholesterol):MUAC (mid-upper arm circumference): N_HDL-c (non-HDL cholesterol): oxLDL (oxidized low-density lipoprotein): TC (total cholesterol): TG (triglycerides): WC (waist circumference): WHR (waist-hip ratio): WHtR (waist circumference-to-height-ratio): and zBMI (BMI z-score).

## Discussion

Obesity is associated with increased prevalence of dyslipidemia and related CV risk [28]. Previous studies have shown that obese children have significantly higher TC, LDL-c, TG, N_HDL-c, and lower HDL-c values [29–31]. With the exception of TC levels, our study results corroborate and agree with these findings. Gender is also an important determinant of CV risk. Kahn *et al* found that girls have higher insulin resistance and TG levels [32]. Likewise, Kumar *et al* [33] and Zhu *et al* [34] reported significantly higher TC and TG levels in girls (8–18 and 7–17 years-old, respectively). The first authors also showed that boys had higher levels of HDL-c, while the second reported a significantly higher mean concentration of LDL-c in girls. These results, with the exception of TC and insulin resistance, agree with our own findings and indicate that these girls may have a higher risk to develop CVD as adults. These differences might be related to how gender affects anthropometric variables.

Some studies suggest that childhood obesity has a considerable short-term and cumulative effect on the cardiovascular system that increases the risk of atherosclerosis in early adult life [35,36]. These effects could be mediated by the relationships between obesity and biochemical parameters, including lipid profile, insulin resistance, and metabolic syndrome. Blüher *et al*, in a cohort of 1.278 children aged 11 to 18 years, found that measures of insulin resistance were most strongly correlated to BMI SD, whereas absolute WC was most strongly associated with other metabolic risk markers, including the lipid profile, though differences in correlation coefficients were minor [37]. However, results of the National Health and Nutrition Examination Survey indicated that WC may be more useful than BMI for identifying adolescents at risk to develop insulin resistance and diabetes in early life [38]. Graves *et al* reported similar associations between these anthropometric measurements at ages 7–9 years and cross-sectional and prospective cardio-metabolic risk factors [39]. In contrast, other studies found WHtR to be more strongly associated with lipid outcomes [40,41]. Nevertheless, our own study data further supports that BMI is more strongly correlated with insulin resistance and the lipid profile than are WC or WHtR.

In addition, our data revealed that oxLDL is significantly and positively correlated with anthropometric parameters (BMI, zBMI, WHtR, and MUAC) and the lipid profile (TC, LDL-c, APO B, ApoB/ApoA1, TC/HDL, LDL/HDL, and N_HDL-c) and negatively correlated with HDL-c levels. Other studies that also explored these correlations reported similar results. In a population of 35 children aged 7–11 years, Islam *et al* demonstrated that oxLDL immune complexes were significantly correlated with TC, LDL-c, and LDL/HDL levels [42]. Kelly *et al* showed that in children ox-LDL is significantly associated with adiposity independent of body fatness [31]. According to Norris *et al*, extreme pediatric obesity is associated with higher levels of oxidative stress and inflammation, suggesting that markers of early CVD and type 2 diabetes mellitus are already present in a population of young children and adolescents [43].

To the best of our knowledge, the present study is the first to describe, in the same population, relationships among extensive anthropometric, bioimpedance, and biochemical parameters, and to evaluate these parameters in a subset of “normal weight” children. Our results showed that according to the analyzed parameters only 29.9% of the “normal weight” children had no CV risk factor. Studies aiming to determine CV risk factors in pediatric populations should take into account the high proportion of children that have an abnormal biochemical parameter. Of note, most previously conducted studies only classified children as being “normal” according to one or two anthropometric or bioimpedance parameters. The findings of this study show that 70.1% of the “normal weight” children had a least one abnormal biochemical parameter and 64.8% had dyslipidemia. Therefore, these results suggest that future studies proposing to assess CV risk in pediatric populations should take into account several anthropometric, bioimpedance, and biochemical parameters to exclude from the “normal” control group those who have one or more parameters outside of the normal range.

The present study also revealed that only 26.6% of overweight and 18.6% obese children lack dyslipidemia, which indicates that children with a higher degree of overweight (defined by BMI) are more likely to have adverse alterations in their lipid profile. In a cohort of 293 adolescents (11–16 years-old), Bibiloni *et al* reported that 48.8% of participants had a positive diagnosis of dyslipidemia and also found that adolescents with a high BMI were more likely to have at least one lipid parameter beyond the normal range, which agrees with our own findings [44].

Several other studies also examined which obesity indexes best predict CV risks markers [32,38,43,44]. However, the relative strength of the associations between several anthropometric measurements and cardio-metabolic risk factors in childhood obesity has not been established. Study cohort size, age range, gender, ethnicity, pubertal stage, absence of internationally accepted classification with age- and gender-specific cut-off values for some anthropometric measures and geographical differences may explain the inconsistent the results. In our current study, we determined the independent contribution of MUAC/WC, WHR (WC/HC) and CC/WC to CVD risk factors in children. Our results suggest that a large HC (adjusted for WC) could be protective for CVD, probably because it assesses skeletal frame size, adipose and muscle mass in the gluteofemoral region, which is positively correlated with leg fat mass and inversely with depots of abdominal visceral and subcutaneous fat [45,46]. The same interpretation applies to MUAC and CC. These results agree with studies of adult populations [47–49]. WC, WHR and WHtR have been used as indicators of abdominal obesity [50] and are associated with adverse CV risk factors. The results of the present study also demonstrate that these parameters, depending on the lipid parameter, are good predictors of a high-risk lipid profile in this population. However, WHR appears to be the better predictor for most lipid variables. Chu *et al* study of 1.366 children (12–16 years-old) concluded that anthropometric parameters (including BMI and WHR) were adequate predictors of blood lipid levels in both genders [51]. Zhu *et al* study of 2.243 school children aged 7–17 years with obesity demonstrated that BMI was better than WC and WHR for identifying dyslipidemia and that the distribution of lipid profiles in Chinese children differ between age groups [34]. According to Agirbasli *et al*, BMI was a better predictor of metabolic syndrome risk variables than skinfold thicknesses, WC, WHtR, and WHR in 9 year old children [52]. However, according to Savva *et al* WC and WHtR were better predictors of CVD risk factors than BMI [40]. The present data suggests that this kind of investigation may have important public health implications because the proposition that anthropometric parameters are good predictors of metabolic risk factors (which might increase the risk of CVD) could justify early interventions.

The study limitations include: 1) the lack of information on the family history of hypercholesterolemia, which according to Kwiterovich [53] explains a large percentage of the variance in lipid concentrations; 2) the cross-sectional nature of the study, which prevents asserting a causal relationship between anthropometric predictors and the lipid profile; and 3) the difficulty in comparing our results with others, which limits external validity.

The primary strengths of this study include the large study population, the comprehensive assessment of surrogate markers of adiposity, some that were never evaluated in children before, and the diverse biochemical parameters. These extensive data have allowed the exploration of many correlations between anthropometric parameters and cardio-metabolic risk factors. Furthermore, we explore the anthropometric measurements as a mean of evaluate the risk for dyslipidemia, and like the precursor *forme fruste* of the osteosarcopenic obesity phenotype in healthy overweight/obese children reported in Stefanaki et al. [54], this dyslipidemia might be a sign of an “in development” earlier form of CVD in young children.

## Conclusion

This study shows that a large proportion of school aged children have at least one abnormality of the lipid profile, including more than half the children of “normal weight” and the great majority of “overweight” and “obese” children. Anthropometric parameters were found to independently predict an altered lipid profile associated with CVD risk. BMI, WC, WHtR and WHR were more directly associated with dyslipidemia, whereas HC was inversely associated. The contribution of each anthropometric measurement to the lipid profile varied with the specific lipid parameter. Selected anthropometric variables are likely to help predict increased odds of having CV risk factors.

## Supporting information

S1 Table Descriptive characteristics of the study population of children by ethnicity. ^§^Age in days were converted in years for a better comparison between groups. ^1^ (Cauc-471/Afr-45/Oth-2). ^2^ (Cauc-458/Afr-50/Oth-8). ^3^ (Cauc-245/Afr-16/Oth-1). Ethnic group means are compared between each characteristic through Bonferroni-adjusted t-tests. Statistical differences are reported as different associated letters: a, b (p < 0.05). APO A1 (apolipoprotein A1): APO B (apolipoprotein B): BMI (body mass index): CC (calf circumference): HC (hip circumference): HDL-c (high-density lipoproteins cholesterol): LDL-c (low-density lipoproteins cholesterol): HOMA-IR (homeostatic model assessment-insulin resistance): MUAC (mid-upper arm circumference): N_HDL-c (non-HDL cholesterol): oxLDL (oxidized low-density lipoprotein): RMR (resting metabolic rate): TC (total cholesterol): TG (triglycerides): WC (waist circumference): WHR (waist-hip ratio): WHtR (waist circumference-to-height-ratio): zBMI (BMI z-score): %BF (percentage body fat): and %SM (percentage skeletal muscle).
(DOCX)

S2 Table Descriptive clinical characteristics of the study population by gender and by IOTF category ^Δ^. ^Δ^According to World Obesity/Policy & Prevention and bioelectrical impedance analysis cut-offs. ^§^Age in days were converted in years for a better comparison between groups. Sex means between each characteristic were compared within each IOTF category while IOTF grade means were compared between each characteristic through Bonferroni-adjusted t-tests. Statistical differences were reported as different associated letters (a.b) or symbols (*.**.***), respectively (p < 0.05). BMI (body mass index): CC (Calf Circumference): HC (hip circumference): MUAC (mid upper arm circumference): RMR (resting metabolic rate): WC (waist circumference): WHR (waist-hip ratio): WHtR (waist circumference-to-height-ratio): zBMI (BMI z-score): %BF (percentage body fat): %BF (percentage body fat): and %SM (percentage skeletal muscle).
(DOCX)

S3 Table Adjusted correlations between biochemical parameters. APO A1 (apolipoprotein A1): APO B (apolipoprotein B): HDL-c (high-density lipoproteins cholesterol): LDL-c (low-density lipoproteins cholesterol): HOMA-IR (homeostatic model assessment-insulin resistance): N_HDL-c (non-HDL cholesterol): oxLDL (oxidized low-density lipoprotein): TC (total cholesterol): TG (triglycerides).
(DOCX)

S4 Table Independent contributions of MUAC and CC to prediction of cardiovascular disease risk factors (adjusted for ethnicity, age, gender and waist circumference). * Not significant. APO A1 (apolipoprotein A1): APO B (apolipoprotein B): CC (calf circumference): HDL-c (high-density lipoproteins cholesterol): LDL-c (low-density lipoproteins cholesterol): MUAC (mid-upper arm circumference): N_HDL-c (non-HDL cholesterol): and oxLDL (oxidized low-density lipoprotein). TC (total cholesterol): TG (triglycerides). (Adverse lipid concentrations of estabilished cut-offs were defined as follows: total cholesterol > 170 mg/dl. LDL-c > 110 mg/dl. HDL-c < 40 mg/dl. triglycerides > 75mg/dl. APO A1 < 1.2. APO B > 0.9 g/L. N_HDL-c ≥ 120. g/L. ox-LDL > 1.38 mU/L).
(DOCX)

S5 Table TRIPOD checklist—prediction model development and validation. *Items relevant only to the development of a prediction model are denoted by D, items relating solely to a validation of a prediction model are denoted by V, and items relating to both are denoted D;V. We recommend using the TRIPOD Checklist in conjunction with the TRIPOD Explanation and Elaboration document.
(DOCX)
