# Supplementary material for: Anthropometric features as predictors of atherogenic dyslipidemia and cardiovascular risk in a large population of school-aged children
Source: PLoS One. 2018 Jun 1;13(6):e0197922. doi: 10.1371/journal.pone.0197922 (PMC5983423; doi:10.1371/journal.pone.0197922)
Supplement: S1 Table — §Age in days were converted in years for a better comparison between groups. 1 (Cauc-471/Afr-45/Oth-2). 2 (Cauc-458/Afr-50/Oth-8). 3 (Cauc-245/Afr-16/Oth-1). Ethnic group means are compared between each characteristic through Bonferroni-adjusted t-tests. Statistical differences are reported as different associated letters: a, b (p < 0.05). APO A1 (apolipoprotein A1): APO B (apolipoprotein B): BMI (body mass index): CC (calf circumference): HC (hip circumference): HDL-c (high-density lipoproteins cholesterol): LDL-c (low-density lipoproteins cholesterol): HOMA-IR (homeostatic model assessment-insulin resistance): MUAC (mid-upper arm circumference): N_HDL-c (non-HDL cholesterol): oxLDL (oxidized low-density lipoprotein): RMR (resting metabolic rate): TC (total cholesterol): TG (triglycerides): WC (waist circumference): WHR (waist-hip ratio): WHtR (waist circumference-to-height-ratio): zBMI (BMI z-score): %BF (percentage body fat): and %SM (percentage skeletal muscle). (DOCX) [file pone.0197922.s001.docx]

**S1 Table. Descriptive characteristics of the study population of children by ethnicity.**

| *Characteristic* | **Caucasian**  (n = 1309) | **African**  (n = 171) | **Others**  (n = 16) |
| --- | --- | --- | --- |
|  | Mean ± SD | Mean ± SD | Mean ± SD |
| Age ^§^ | 9.71_a_ ± 0.6 | 9.9_b_ ± 0.7 | 9.79_a.b_ ± 0.5 |
| ***Anthropometry*** | | | |
| Weight (Kg) | 35.6_a_ ± 8.8 | 36.3_a_ ± 8.87 | 34.6_a_ ± 7.51 |
| Height (cm) | 138.0_a_ ± 7.3 | 141.1_b_ ± 7.8 | 37.1_a.b_ ± 7.5 |
| BMI (Kg/m^2^) | 18.5_a_ ± 3.27 | 18.1_a_ ± 3.28 | 8.3_a_ ± 2.93 |
| zBMI | 0.69_a_ ± 1.07 | 0.46_b_ ± 1.2 | 0.63_a.b_ ± 0.94 |
| WC (cm) | 65.5_a_ ± 9.28 | 63.5_b_ ± 8.64 | 63.4_a.b_ ± 6.95 |
| HC (cm) | 72.0_a_ ± 7.8 | 71.2_a_ ± 9.1 | 69.3_a_ ± 8 |
| WHR (WC/HC) | 0.89_a_ ± 0.05 | 0.88_a_ ± 0.05 | 0.91_a_ ± 0.04 |
| WHtR (WC/height) | 0.47_a_ ± 0.06 | 0.45_b_ ± 0.054 | 0.46_a.b_ ± 0.04 |
| MUAC (cm) | 21.2_a_ ± 2.9 | 21.2_a_ ± 2.9 | 20.4_a_ ± 2.3 |
| CC (cm) | 29.2_a_ ± 3.4 | 28.6_a_ ± 3.3 | 28.2_a_ ± 3 |
| ***Bioelectrical impedance*** | | | |
| BF (%) | 22.2_a_ ± 7.7 | 20.2_b_ ± 8.3 | 20.8_a.b_ ± 5.5 |
| SM (%) | 31.8_a_ ± 2.8 | 32.6_b_ ± 2.7 | 31.3_a.b_ ± 2.5 |
| RMR (Kcal/day) | 1211_a_ ± 117 | 1219_a_ ± 110 | 1170_a_ ± 94 |
| ***Biochemical Parameters*** | | | |
| Glycemia (mg/dl) | 77.8_a_ ± 10.7 | 78.7_a_ ± 11.1 | 78.3_a_ ± 13.4 |
| TC (mg/dl) | 170.4_a_ ± 29 | 170.5_a_ ± 33.1 | 182.5_a_ ± 26.2 |
| LDL-c (mg/dl) | 94.0_a_ ± 23.9 | 93.2_a_ ± 28.3 | 102.3_a_ ± 25.8 |
| HDL-c (mg/dl) | 56.4_a_ ± 11.2 | 56.2_a_ ± 11.1 | 58.7_a_ ± 13.3 |
| TG (mg/dl) | 62.7_a_ ± 27.1 | 59.5_a_ ± 24.3 | 75.5_a_ ± 28.8 |
| APO A1(g/L) | 1.36_a_ ± 0.19 | 1.34_a_ ± 0.19 | 1.41_a_ ± 0.15 |
| APO B (g/L) | 0.74_a_ ± 0.16 | 0.72_a_ ± 0.17 | 0.88_b_ ± 0.3 |
| APO B/APO A1 | 0.6_a_ ± 0.1 | 0.5_a_ ± 0.1 | 0.6_a_ ± 0.2 |
| LDL-c/Apo B | 1.27_a_± 1.5 | 1.27_a_ ± 1.7 | 1.23_a_ ± 2.7 |
| TC/HDL | 3.1_a_ ± 0.6 | 3.1_a_ ± 0.7 | 3.2_a_ ± 0.8 |
| N_ HDL-c (mg/dl) | 114_a_ ± 26 | 114_a_ ± 30 | 124_a_ ± 23 |
| LDL/HDL | 1.73_a_ ± 0.55 | 1.72_a_ ± 0.6 | 1.82_a_ ± 0.59 |
| Total Proteins (mg/dl) | 7.28_a_ ± 0.7 | 7.42_b_ ± 0.7 | 7.46_a.b_ ± 0.8 |
| Ferritin (ng/ml) | 39.4_a_ ± 20.6 | 36.3_a_ ± 22.1 | 52.5_b_ ± 55.8 |
| Creatinine (mg/dl) | 0.59_a_ ± 0.1 | 0.61_a_ ± 0.1 | 0.6_a_ ± 0.1 |
| Insulin (µU/ml)^1^ | 7.1_a_ ± 11.15 | 9.48_a_ ± 15.48 | 4.24_a_ ± 0.15 |
| Homa-IR | 1.38_a_ ± 2.43 | 1.97_a_ ± 3.45 | 0.75_a_ ± 0.04 |
| Leptin (ng/ml)^2^ | 10.9_a_ ± 10.7 | 10.9_a_ ± 13.3 | 14.9_a_ ± 9.6 |
| oxLDL (mU/l)^3^ | 6.6_a_ ± 1.8 | 6.3_a_ ± 2.0 | 6.6 _a_ |

^§^Age in days were converted in years for a better comparison between groups. ^1^(Cauc-471/Afr-45/Oth-2). ^2^(Cauc-458/Afr-50/Oth-8). ^3^(Cauc-245/Afr-16/Oth-1). Ethnic group means are compared between each characteristic through Bonferroni-adjusted t-tests. Statistical differences are reported as different associated letters: a, b (p < 0.05). APO A1 (apolipoprotein A1): APO B (apolipoprotein B): BMI (body mass index): CC (calf circumference): HC (hip circumference): HDL-c (high-density lipoproteins cholesterol): LDL-c (low-density lipoproteins cholesterol): HOMA-IR (homeostatic model assessment-insulin resistance): MUAC (mid-upper arm circumference): N_HDL-c (non-HDL cholesterol): oxLDL (oxidized low-density lipoprotein): RMR (resting metabolic rate): TC (total cholesterol): TG (triglycerides): WC (waist circumference): WHR (waist-hip ratio): WHtR (waist circumference-to-height-ratio): zBMI (BMI z-score): %BF (percentage body fat): and %SM (percentage skeletal muscle).
