# Supplementary material for: Anthropometric features as predictors of atherogenic dyslipidemia and cardiovascular risk in a large population of school-aged children
Source: PLoS One. 2018 Jun 1;13(6):e0197922. doi: 10.1371/journal.pone.0197922 (PMC5983423; doi:10.1371/journal.pone.0197922)
Supplement: S2 Table — ΔAccording to World Obesity/Policy & Prevention and bioelectrical impedance analysis cut-offs. §Age in days were converted in years for a better comparison between groups. Sex means between each characteristic were compared within each IOTF category while IOTF grade means were compared between each characteristic through Bonferroni-adjusted t-tests. Statistical differences were reported as different associated letters (a.b) or symbols (*.**.***), respectively (p < 0.05). BMI (body mass index): CC (Calf Circumference): HC (hip circumference): MUAC (mid upper arm circumference): RMR (resting metabolic rate): WC (waist circumference): WHR (waist-hip ratio): WHtR (waist circumference-to-height-ratio): zBMI (BMI z-score): %BF (percentage body fat): %BF (percentage body fat): and %SM (percentage skeletal muscle). (DOCX) [file pone.0197922.s002.docx]

|  | Normal | | | Overweight | | | Obese | | |
| --- | --- | --- | --- | --- | --- | --- | --- | --- | --- |
| *Characteristic* | Total  (n = 1071) | Male  (n=523) | Female  (n=548) | Total  (n = 312) | Male  (n=140) | Female  (n=172) | Total  (n = 113) | Male  (n=60) | Female  (n=53) |
|  |  |  |  |  |  |  |  |  |  |
|  | Mean ± SD | Mean ± SD | Mean ± SD | Mean ± SD | Mean ± SD | Mean ± SD | Mean ± SD | Mean ± SD | Mean ± SD |
| Age^§^ | 9.75* ± 0.59 | 9.75_a_ ± 0.61 | 9.75_a_ ± 0.56 | 9.68* ± 0.26 | 9.82_b_ ± 0.76 | 9.57_a_ ± 0.62 | 9.75* ± 0.26 | 9.73_a_ ± 0.51 | 9.78_a_ ± 0.53 |
| Weight (Kg) | 31.8* ± 5.4 | 31.9_a_ ± 5.4 | 31.6_a_ ± 5.47 | 42.3** ± 5.5 | 42.8_a_ ± 6.1 | 41.9_a_ ± 4.9 | 53.1*** ± 7.7 | 53.2_a_ ± 7.5 | 52.97_a_ ± 8.0 |
| Height (cm) | 137.2* ± 7.4 | 137.6_a_ ± 7.1 | 136.9_a_ ± 7.6 | 140.6** ± 6.6 | 141.4_b_ ± 7.0 | 140.0_a_ ± 6.2 | 143.4*** ± 6.6 | 143.2_a_ ± 5.9 | 143.6_a_ ± 7.3 |
| BMI (Kg/m^2^) | 16.7* ± 1.6 | 16.7_a_ ± 1.5 | 16.8_a_ ± 1.7 | 21.3** ± 1.3 | 21.3_a_ ± 1.3 | 21.3_a_ ± 1.3 | 25.7*** ± 2.2 | 25.8_a_ ± 2.1 | 25.6_a_ ± 2.2 |
| zBMI | 0.12* ± 0.8 | 0.12_a_ ± 0.8 | 0.13_a_ ± 0.8 | 1.7** ± 0.3 | 1.8_a_ ± 0.3 | 1.7_a_ ± 0.3 | 2.6*** ± 0.3 | 2.6_b_ ± 0.3 | 2.5_a_ ± 0.2 |
| WC (cm) | 61.1* ± 5.4 | 60.1_a_ ± 4.9 | 61.3_a_ ± 5.9 | 72.3** ± 5.3 | 72.6_a_ ± 5.3 | 72.1_a_ ± 5.3 | 83.6*** ± 9.1 | 82.4_a_ ± 9.8 | 85.1_a_ ± 8.0 |
| HC (cm) | 68.2* ± 5.6 | 67.6_b_ ± 5.2 | 68.9_a_ ± 5.8 | 78.6**± 4.7 | 78.3_a_ ± 4.7 | 78.8_a_ ± 4.7 | 86.0*** ± 6.5 | 85.6_a_ ± 6.1 | 86.7_a_ ± 7.1 |
| WHR (WC/HC) | 0.88* ± 0.05 | 0.89_b_ ± 0.05 | 0.88_a_ ± 0.05 | 0.91** ± 0.05 | 0.92_b_ ± 0.04 | 0.90_a_ ± 0.05 | 0.94*** ± 0.06 | 0.93_a_ ± 0.07 | 0.95_a_ ± 0.05 |
| WHtR (WC/height) | 0.45* ± 0.03 | 0.44_b_ ± 0.03 | 0.45_a_ ± 0.04 | 0.52** ± 0.03 | 0.52_a_ ± 0.03 | 0.52_a_ ± 0.04 | 0.59*** ± 0.06 | 0.58_a_ ± 0.06 | 0.6_a_ ± 0.05 |
| MUAC (cm) | 19.9* ± 1.9 | 19.7_b_ ± 1.8 | 20.1_a_ ± 1.9 | 23.7** ± 1.6 | 23.8_a_ ± 1.7 | 23.7_a_ ± 1.5 | 26.4*** ± 2.4 | 26.6_a_ ± 2.5 | 26.1_a_ ± 2.3 |
| CC (cm) | 27.7* ± 2.3 | 27.8_a_ ± 2.2 | 27.7_a_ ± 2.4 | 31.6** ± 2.2 | 31.8_a_ ± 2.2 | 31.5_a_ ± 2.2 | 35.2*** ± 3.2 | 35.4_a_ ± 2.7 | 34.9_a_ ± 3.6 |
| BF (%) | 18.2* ± 5.3 | 17.8_b_ ± 4.96 | 18.55_a_ ± 5.48 | 29.0** ± 3.5 | 28.0_b_ ± 3.16 | 29.7_a_ ± 3.6 | 36.0*** ± 3.4 | 35.43_b_ ± 3.2 | 36.77_a_ ± 3.5 |
| SM (%) | 32.3*± 3.0 | 32.83_b_ ± 3.0 | 31.7_a_ ± 2.8 | 31.4** ± 2.0 | 32.55_b_ ± 1.9 | 30.46_a_ ± 1.5 | 29.8*** ± 2.0 | 30.81_b_ ± 1.6 | 28.68_a_ ± 1.7 |
| RMR (Kcal/day) | 1166* ± 86 | 1191_b_ ± 85 | 1142_a_ ± 78 | 1288** ± 81 | 1338_b_ ± 82 | 1249_a_ ± 55 | 1406*** ±117 | 1464_b_ ± 117 | 1338_a_ ± 74 |

**S2 Table: Descriptive clinical characteristics of the study population by gender and by IOTF category ^∆^.**

^∆^According to World Obesity/Policy & Prevention and bioelectrical impedance analysis cut-offs. ^§^Age in days were converted in years for a better comparison between groups. Sex means between each characteristic were compared within each IOTF category while IOTF grade means were compared between each characteristic through Bonferroni-adjusted t-tests. Statistical differences were reported as different associated letters (a.b) or symbols (*.**.***), respectively (p < 0.05). BMI (body mass index): CC (Calf Circumference): HC (hip circumference): MUAC (mid upper arm circumference): RMR (resting metabolic rate): WC (waist circumference): WHR (waist-hip ratio): WHtR (waist circumference-to-height-ratio): zBMI (BMI z-score): %BF (percentage body fat): %BF (percentage body fat): and %SM (percentage skeletal muscle).
