# Supplementary material for: Anthropometric features as predictors of atherogenic dyslipidemia and cardiovascular risk in a large population of school-aged children
Source: PLoS One. 2018 Jun 1;13(6):e0197922. doi: 10.1371/journal.pone.0197922 (PMC5983423; doi:10.1371/journal.pone.0197922)
Supplement: S3 Table — APO A1 (apolipoprotein A1): APO B (apolipoprotein B): HDL-c (high-density lipoproteins cholesterol): LDL-c (low-density lipoproteins cholesterol): HOMA-IR (homeostatic model assessment-insulin resistance): N_HDL-c (non-HDL cholesterol): oxLDL (oxidized low-density lipoprotein): TC (total cholesterol): TG (triglycerides). (DOCX) [file pone.0197922.s003.docx]

|  | Total Proteins (mg/dl) | Ferritin (ng/ml) | Creatinine (mg/dl) | Leptin (ng/ml) | oxLDL (mU/L) | oxLDL/  LDL |
| --- | --- | --- | --- | --- | --- | --- |
| Glycemia (mg/dl) | 0.015 | -0.005 | 0.071 | 0.137 | 0.085 | 0.042 |
| Insulin (mU/L) | -0.047 | -0.033 | 0.075 | 0.205 | 0.093 | -0.072 |
| Homa-IR | -0.058 | -0.028 | 0.124 | 0.198 | 0.102 | -0.058 |
| TC (mg/dl) | 0.310 | 0.073 | 0.069 | 0.079 | 0.639 | -0.146 |
| LDL-c (mg/dl) | 0.159 | 0.097 | 0.003 | 0.196 | 0.696 | -0.362 |
| HDL-c (mg/dl) | 0.054 | -0.007 | 0.028 | -0.175 | -0.044 | 0.027 |
| TG (mg/dl) | 0.165 | 0.042 | 0.033 | 0.353 | 0.300 | -0.078 |
| APO A1 (g/L) | 0.084 | -0.004 | 0.032 | -0.130 | 0.063 | 0.073 |
| APO B (g/L) | 0.251 | 0.099 | 0.024 | 0.152 | 0.602 | -0.184 |
| APO B/APO A1 | 0.160 | 0.077 | -0.001 | 0.184 | 0.501 | -0.188 |
| LDL-c/Apo B | -0.076 | 0.026 | -0.044 | 0.100 | 0.169 | -0.240 |
| TC/HDL | 0.217 | 0.074 | 0.033 | 0.248 | 0.580 | -0.157 |
| LDL/HDL | 0.097 | 0.087 | -0.015 | 0.271 | 0.573 | -0.302 |
| N_ HDL-c (mg/dl) | 0.318 | 0.079 | 0.064 | 0.156 | 0.700 | -0.166 |

**S3 Table: Adjusted correlations between biochemical parameters.**

APO A1 (apolipoprotein A1): APO B (apolipoprotein B): HDL-c (high-density lipoproteins cholesterol): LDL-c (low-density lipoproteins cholesterol): HOMA-IR (homeostatic model assessment-insulin resistance): N_HDL-c (non-HDL cholesterol): oxLDL (oxidized low-density lipoprotein): TC (total cholesterol): TG (triglycerides).
