# Supplementary material for: Anthropometric features as predictors of atherogenic dyslipidemia and cardiovascular risk in a large population of school-aged children
Source: PLoS One. 2018 Jun 1;13(6):e0197922. doi: 10.1371/journal.pone.0197922 (PMC5983423; doi:10.1371/journal.pone.0197922)
Supplement: S4 Table — * Not significant. APO A1 (apolipoprotein A1): APO B (apolipoprotein B): CC (calf circumference): HDL-c (high-density lipoproteins cholesterol): LDL-c (low-density lipoproteins cholesterol): MUAC (mid-upper arm circumference): N_HDL-c (non-HDL cholesterol): and oxLDL (oxidized low-density lipoprotein). TC (total cholesterol): TG (triglycerides). (Adverse lipid concentrations of estabilished cut-offs were defined as follows: total cholesterol > 170 mg/dl. LDL-c > 110 mg/dl. HDL-c < 40 mg/dl. triglycerides > 75mg/dl. APO A1 < 1.2. APO B > 0.9 g/L. N_HDL-c ≥ 120. g/L. ox-LDL > 1.38 mU/L). (DOCX) [file pone.0197922.s004.docx]

|  | | | | | | | | |
| --- | --- | --- | --- | --- | --- | --- | --- | --- |
| Odds ratio (95% confidence interval) | | | | | | | | |
|  | TC  (mg/dl) | LDL-c  (mg/dl) | HDL-c  (mg/dl) | TG  (mg/dl) | Apo A1  (g/L) | Apo B  (g/L) | N_HDL-c  (mg/dl) | oxLDL  (mU/L) |
| MUAC/WC | 2.099  (0.285 – 15.475)* | 7.284  (0.118 – 451.001)* | 0.001  (0.000 – 338.38)* | 0.036  (0.000 -10.331)* | 0.001  (0.000-0.684 ) | 0.345  ( 0.001-93.96 )* | 2.210  (0.4- 12.222)* | 41620  (0.003- 6.1E+11)* |
| CC/WC | 0.674  (0.046 – 9.958)* | 0.057  (0.001 – 2.226)* | 0.000  (0.000 – 0.028) | 0.001  (0.000 – 0.071) | 0.02  (0.00-1.307)* | 0.012  (0.00 -1.409)* | 0.009  (0.000 – 0.268) | 0.034  (0.000 – 4254.429)* |
|  | | | | | | | | |

**S4 Table. Independent contributions of MUAC and CC to prediction of cardiovascular disease risk factors (adjusted for ethnicity, age, gender and waist circumference).**

* Not significant. APO A1 (apolipoprotein A1): APO B (apolipoprotein B): CC (calf circumference): HDL-c (high-density lipoproteins cholesterol): LDL-c (low-density lipoproteins cholesterol): MUAC (mid-upper arm circumference): N_HDL-c (non-HDL cholesterol): and oxLDL (oxidized low-density lipoprotein). TC (total cholesterol): TG (triglycerides). (Adverse lipid concentrations of estabilished cut-offs were defined as follows: total cholesterol > 170 mg/dl. LDL-c > 110 mg/dl. HDL-c < 40 mg/dl. triglycerides > 75mg/dl. APO A1 < 1.2. APO B > 0.9 g/L. N_HDL-c ≥ 120. g/L. ox-LDL > 1.38 mU/L).
